# Advancing the Science of Patient Input in Drug Research and Development

**DOI:** 10.2196/74436

**Published:** 2026-05-01

**Authors:** Marilyn A Metcalf, Henrietta Awo Osei-Anto, Thomas J Sharpe, Luther T Clark, Carolyn K Shore

**Affiliations:** 1 GSK Durham, NC United States; 2 Tiwaa Health Consultancy Washington, DC United States; 3 Sharpe Patient Insights Fort Lauderdale, FL United States; 4 Merck New Jersey, NJ United States; 5 National Academies of Sciences, Engineering, and Medicine Washington, DC United States

**Keywords:** clinical trials, clinical research, drug research and development, patient input, patient-centered

## Abstract

The integration of patient input into drug research & development (R&D) enables the production of medicines that better meet the needs of patients. While momentum for advancing the science of patient input has continued to grow, there remain a host of barriers to full implementation and integration of systematic approaches for collecting and using robust and meaningful patient input data to inform decision-making. To help address these barriers, the Advancing the Science of Patient Input Action Collaborative (the collaborative), an activity associated with the National Academies of Sciences, Engineering, and Medicine (National Academies) Forum on Drug Discovery, Development, and Translation, organized a multi-stakeholder endeavor to identify and address key barriers to implementation through a series of information-gathering efforts. The collaborative engaged a wide range of perspectives to seek out practical paths forward to better align drug discovery, development, and regulation with patient priorities for disease management and treatment. Collaborative participants focused on three overarching research priorities which, if effectively addressed, would help advance the science of patient input: 1) understanding the patient experience over the course of a given disease or medical condition, 2) capturing the patient perspectives and priorities on benefit-risk, and 3) incorporating patient input into clinical trial design and continuous improvement. Addressing these research priorities would help decision makers shift away from using patient input in particular cases or for one-off applications and towards the integration of patient input as part of everyday medical research and practice. Building upon existing guidances and strategies, and sharing lessons learned from use cases, a comprehensive patient input framework would serve as a critical step towards reimagining and enriching the science of patient input throughout the drug R&D process, enabling a future in which medicines more fully meet the needs of patients.

## Introduction

As experts in their own experience, people living with a disease or condition play a critical role in informing drug research and development (R&D). The growing momentum for incorporating patient input throughout the drug R&D life cycle—from early-stage drug discovery to clinical trial design and execution—builds upon the foundational principles of patient and public involvement in health research. This approach provides an opportunity to transform traditionally anecdotal and qualitative patient input into rigorous, credible evidence that is scientifically robust and, in some cases, suitable for regulatory decision-making. The science of patient input is defined in the Food and Drug Administration (FDA) Patient-Focused Drug Development (PFDD) Glossary as “methods and approaches of systematically obtaining, analyzing, and using information that captures patients’ experiences, perspectives, needs, and priorities in support of the development and evaluation of medical products.” In addition to regulators, pharmaceutical companies increasingly rely upon patient input to help inform scientific and regulatory decisions in drug R&D as well as clinical operations.

For purposes of this paper, patient input refers to information, perspectives, experiences, preferences, and priorities that patients might provide to inform scientific or regulatory decision-making. The concept is distinct from patient engagement, defined by the FDA as “intentional, meaningful interactions with patients that provide opportunities for mutual learning and effective collaborations,” which focuses more on how patients participate in the R&D process.

Application of the science of patient input can help clarify patient perspectives on benefit-risk and outcome priorities, informing clinical trial design and supporting regulatory decision-making. One clear case is the effort by regulators to offer strategic, regulatory, programmatic, and policy assistance to facilitate the incorporation of patient input into R&D decision-making. The FDA’s PFDD Program within the Center for Drug Evaluation and Research provides opportunities to gather patient insights on disease experiences and treatments. PFDD meetings are convened directly by the FDA and by external groups, helping to inform FDA guidance development. For example, a 2019 guidance on drug development for amyotrophic lateral sclerosis encourages drug developers to develop new outcome measures using patient experience data that can assess clinically meaningful effects in patients [1].

Based on work of the Advancing the Science of Patient Input Action Collaborative (the collaborative), an ad hoc activity associated with the National Academies of Sciences, Engineering, and Medicine’s Forum on Drug Discovery, this paper seeks to fill the gap between strategic or conceptual frameworks for patient input and scaled operationalization such that the science of patient input is embedded in decision-making across the R&D life cycle. Through a structured process of convenings, surveys, and cross-sector dialogue, the collaborative brought together people working in academia, industry, regulatory agencies, and health care, and people with lived experience to identify practical barriers to implementation and outline research priorities for operationalizing the use of patient input in drug R&D. This paper draws on the collaborative’s work, sets it in broader context of patient input literature, and outlines practical steps to enable the systematic study and development of validated patient-centered methodologies—where “patient-centered” refers to approaches that prioritize patients’ needs, preferences, values, and experiences—to ensure that patient input is rigorously collected, measured, and integrated into the drug development ecosystem.

## Activities

To encourage more transparent agreement across stakeholders on the incorporation of patient input into decision-making for drug development and use, the collaborative gathered people with leadership roles and expertise in drug and device R&D, drug regulation, and patient research and engagement. Participants began their work by (1) cataloguing current efforts to advance the science of patient input, (2) identifying and prioritizing critical gaps in the knowledge base and other barriers that impede progress, and (3) laying out next steps to help advance the field.

To better understand some of the gaps and opportunities in the science of patient input, collaborative participants undertook the following activities:

1. Stakeholder questionnaire: To identify ongoing efforts, perceived gaps, and implementation barriers, collaborative participants circulated questions to 8 individuals (3 respondents) and 17 organizations (5 respondents) representing regulatory agencies, biopharmaceutical companies, patient groups, and vendors. Respondents were offered the option of responding in writing or through informal phone interviews. In addition, The Professional Society for Health Economics and Outcomes Research circulated questions to 6 relevant member special interest groups (30 respondents).
2. Public workshop: Titled “Advancing the Science of Patient Input in Medical Product R&D: Towards a Research Agenda,” featuring facilitated discussions and identification of themes [2].
3. Cross-sector collaborative meeting: Convened participants to discuss research priorities and opportunities to advance the science of patient input (Multimedia Appendix 1).

Early discussions covered all medical products, including drugs, biologics, and devices, each of which comes with a different set of considerations for risk and benefit for patients as well as different approaches for demonstrating safety and efficacy, timelines for development, and the regulatory review processes. Developers and regulators of medical devices have taken steps to incorporate end user input, experience, and testing (eg, practitioners operating a device or patients using a device in the home setting) into development and approval processes. In contrast, drug development and approval processes have taken longer to incorporate such approaches into “business as usual” in the preclinical and clinical settings. For this reason, collaborative participants focused on challenges and opportunities for integrating patient input into drug R&D at scale.

## Findings

### Challenges and Opportunities for Integrating Patient Input Into Drug R&D

One gap highlighted by the collaborative participants is the lack of information and insights collected from patient populations that are representative of those who live with a given disease. It is important to understand which aspects of disease burden and treatment matter most to patients with the condition a given drug is intended to treat. Patient organizations, electronic health records (EHRs), and online patient communities offer fruitful opportunities to gather insights. However, the majority of patients are not affiliated with a patient group, do not participate in research, and may not be connected to health care institutions participating in clinical trials. This creates a void in what information is collected and leads to omissions or inaccurate assumptions that filter their way from initial information gathering through integration of patient-centric studies and tools.

To advance the science of patient input, that gap in data must be filled. Patient input should be collected from across representative patient populations with consideration for age, sex, socioeconomic status, and disease severity, among other relevant attributes that may impact how a drug could perform. Approaches to reach broad patient populations through traditional and nontraditional means will become increasingly important given changes to the health care landscape, variable access to health care, the emergence or resurgence of infectious diseases, and the abundance of health misinformation.

### Research Priorities for Advancing the Science of Patient Input

To focus efforts on the integration of patient input where it would have the greatest value and impact, collaborative participants assessed information gathered from wide-ranging stakeholders and distilled it to three research priorities:

1. Understanding the patient experience over the course of a disease or condition: Clinical researchers and sponsors cannot fully meet patient needs until they are able to consistently collect and use meaningful and holistic patient experience data. An understanding of a patient’s baseline, meaningful difference, disease trajectory, health care disparities, and psychological burden requires accurate data and thorough evaluation. However, data on the full course of a disease from a patient’s perspective are not adequately captured in health records or through available research instruments. If achieved, a view of the full longitudinal patient journey could better enable PFDD that prioritizes interventions that maximize meaningful outcomes for patients.
2. Capturing patient perspectives and priorities regarding the benefits and risks of medical product use: Despite the critical importance of patient input on benefits and risks, patients’ views are not measured accurately, nor applied with the same weight given to that of other stakeholders. Patients may value certain outcomes or tolerability profiles very differently from clinicians or regulators. Additionally, patients’ perspectives on benefits and risks may differ depending upon the nature and progression of their disease, their life stage, comorbidities, or cultural background. Standard tools and measures do not adequately capture these important nuances, leading to conclusions that may not accurately reflect the patient population.
3. Incorporating patient input into continuous improvement of clinical trial design and development: The process of refining and optimizing clinical trial design and development would benefit from the incorporation of patient input. However, several gaps continue to impede progress in this area, including a lack of consistent practices for the collection and use of patient input to support continuous improvement; inadequate measures for assessing the impact of clinical trial participation on patients, caregivers, and families; and a lack of robust metrics for demonstrating the effect of patient input on protocol design, as well as clinical trial enrollment and retention.

The participants then examined other crosscutting barriers preventing the science of patient input from being used to its full potential (Textbox 1).

**Barriers to advancing the science of patient input identified in collaborative participant questionnaire responses.**

**Understanding the patient experience with a disease or a medical condition may be hampered by the following barriers**

- Lack of data capturing the full patient experience, including both personal and clinical experience.
- Patient perspectives input are not adequately incorporated into electronic health records.
- Existing patient-reported outcomes and patient-centered outcomes may not reflect baseline status or meaningful difference for individual patients.
- Difficulty incorporating learnings from patient input into care and research systems to inform new treatment development (ie, learning health systems and care-based research).
- Lack of defined quality standards and rigor for both quantitative and qualitative patient input data.
- Clinical data collection often does not generate information that provides a holistic view of the patient.
- Symptom burden from the perspective of the patient that maps to disease trajectory is not captured in a standardized manner.
- Databases not designed to collect or integrate patient input.
- Missing voices in available data.
- Difficulty reaching or engaging certain patient populations, particularly those with rare diseases.
- Limited patient input on health care disparities.
- Need for methods and metrics to quantify and measure psychological burden of disease on patients.

**Capturing patient perspectives and preferences on benefit-risk may be hampered by the following barriers**

- Need for better understanding of how patients perceive risk compared with other stakeholders (eg, pharmaceutical companies, clinicians, and regulators).
- Lack of methodologies for quantifying change in benefit-risk tolerance or uncertainty tolerance over time across age, or with disease progression.
- Lack of methodologies for obtaining input from patients unable to directly express preferences (eg, pediatric patients and cognitively impaired patients, such as those with dementia or Alzheimer disease).
- Insufficient knowledge regarding how patients with different diseases or conditions cope with uncertainty.
- Underrepresentation of perspectives from patients with multiple comorbidities.
- Limited understanding and methods for institutional review boards to incorporate patient perspectives.

**Incorporating patient input in clinical trial development and continuous improvement may be hampered by the following barriers**

- Need for approaches that encourage a culture of incorporating patient input early in trial design.
- Lack of metrics to assess value of patient input in protocol development.
- Lack of training for researchers on the value and use of patient input.
- Insufficient methods for understanding how different patient populations perceive and interpret information due to cultural or social differences.
- Challenge in defining and validating meaningful measures.
- Limited training for patients and patient groups to provide input effectively.
- Need for methods to better represent the broader patient community.
- Lack of approaches for capturing caregiver and family experiences throughout the clinical trial process.
- Lack of understanding and methods to evaluate how patient input affects trial enrollment and retention.

#### Research Priority: Understanding the Patient Experience Over the Course of a Disease or Condition

Given these challenges, collaborative participants suggested actions to address the barriers (Multimedia Appendix 2) along with benefits of taking those actions from the perspectives of a number of stakeholders (Multimedia Appendix 3). Building on this body of work, collaborative participants considered the need for approaches laid out in the following sections.

##### A Comprehensive Framework to Elucidate the Patient Experience and Capture Burden as Diseases Progress Over Time

The meaningful collection, quantification, and assessment of disease or treatment burden over time would enable stakeholders to better demonstrate measurable improvements in patient care, including quality of life and other nonmedical outcomes, throughout the patient journey. Evaluation of patient experience throughout diagnosis, treatment, and recovery periods would enable researchers, drug developers, and regulators to better understand and address benefit-risk for therapies from the perspective of the patient.

##### Mechanisms to Identify and Validate Methodologies and Metrics for Incorporating Relevant Patient Perspectives Into EHRs

EHRs already capture patient information over time. Merging EHRs alongside other technologies (eg, electronic data capture, digital health technologies, artificial intelligence, and machine learning) would enable a more comprehensive view of disease experience. However, there are currently few seamless ways to account for the myriad variables that affect health and quality of life during a patient’s treatment journey. Combining disparate sources of patient information (eg, patient-reported outcomes and digital health technologies) could further validate patient observation and input, supporting a more evidence-based approach for informing clinical trials and improving patient care [3]. These types of approaches require pilot testing to determine practical applications for clinicians, patients, families, and caregivers, while avoiding undue burden. The use of frameworks for best practices in clinical outcome assessments may be one approach for demonstrating how EHRs and clinical trial data can be combined to better understand the patient experience.

#### Research Priority: Capturing Patient Perspectives and Priorities on Benefits and Risks of Medicine Use

An individual’s understanding of the benefits and risks of medicines is critical to informing their decision-making process around participation in research and clinical care [4]. More broadly, improving people’s understanding of the benefits and risks of engaging in clinical research could help empower potential trial participants to make better-informed treatment decisions [5]. Capturing patient perspectives and priorities on benefit and risk could also help inform drug development and facilitate shared decision-making in ways that are beneficial for patients and their caregivers.

Stakeholders can apply core principles for developing and incorporating effective measurements that are meaningful to patients, such as those recommended in the Medical Device Innovation Consortium Patient-Centered Benefit-Risk Framework and the European Organisation for Research and Treatment of Cancer Quality of Life Questionnaire C30 [6,7].

The FDA and other key stakeholders have laid out guidance and frameworks on how to develop generalizable approaches and tools to capture and improve understanding of benefit-risk for trial participants [8,9].

While these approaches are helpful as a start, collaborative participants identified numerous opportunities for further refinement to provide more holistic information that would reflect patients’ experiences and support more informed decision-making (Table S1 in Multimedia Appendix 4). Through a questionnaire, a public National Academies workshop, and collaborative discussions, participants described types of patient input for capturing patient perspectives and priorities on benefits and risks of medicine use, methods and data sources to collect the input, gaps or barriers to implementing the patient input, potential research approaches to promote use of the patient input, and relevant stakeholders.

Building on this body of work, collaborative participants considered the following approaches: identify and validate metrics to better understand how certain populations perceive and understand information regarding risks and benefits differently due to a host of overlapping factors (eg, social, cultural, accessibility, geography, disease prognosis).

Patient views and understanding of benefit and risk may vary significantly based on several variables, including disease presentation and progression, treatment effect, standard of care, quality of life, comorbidities, and social determinants of health. Establishing metrics that reflect the range of patient experiences and perspectives across different populations would help decision makers better design and implement clinical trials that meet the needs of patients.

##### Identify and Validate Metrics for Obtaining Input From Patient Populations With Multiple Comorbidities

Many clinical trials exclude large segments of the population, including those living with common chronic diseases—cardiometabolic diseases, dementia, cancer, or psychiatric conditions. While exclusion criteria can better enable the assessment of safety and efficacy of a medical product under controlled conditions, this can limit the relevance of clinical trial results for the range of people who will use the product once it has been approved.

Future approaches may include the development of metrics for obtaining patient input on benefit and risk that includes people who are living with commonly occurring comorbid conditions, a lesson illustrated by COVID-19 clinical trials. Early studies showed that the presence of comorbidities, including cardiovascular diseases, autoimmune conditions, and metabolic disorders (eg, diabetes and obesity) was associated with higher levels of patient hospitalization, disease severity and mortality, and risk for long COVID symptoms [10-12]. However, up to half of COVID-19 treatment trials initiated in 2020 in the United States excluded patients with high-risk chronic conditions. This may have obscured insights on outcomes for relevant patient populations that were disproportionately affected by COVID-19 [13]. Resolving the discordance between clinical trials and real-world applications could improve patient outcomes by illuminating subpopulation-specific stratification of therapeutic approaches.

##### Identify and Validate Metrics to Integrate Patient Input for Institutional Review Boards

Frameworks for comprehensive patient input are needed to ensure that representative patient perspectives are considered in institutional review board determinations. Rather than relying on input from 1 patient or community member serving on the institutional review board, a road map for capturing perspectives for participants of varying age, race, ethnicity, geography, gender, socioeconomic status, education, stage of disease, and insurance statuses is more desirable. This road map may be presented by “patient experts,” individuals who not only identify as patients but also serve as advocates for the broader patient population. A tangible output of “patient expert” voices could take the form of ethics rubrics that incorporate patient input throughout the diagnosis, treatment, and recovery timelines.

#### Research Priority: Incorporating Patient Input Into Continuous Improvement of Clinical Trial Design and Development

Patient-centered approaches and metrics for clinical trial design are vital for improving participant experience throughout the drug R&D life cycle (Table S1 in Multimedia Appendix 5). Patient input during trial design and early clinical studies can provide insights into unmet patient needs, side effect tolerability, and treatment preferences, as well as informing recruitment and retention strategies [14-16]. Phase III trials using patent engagement practices are more likely to reach approval status than studies that were not patient-centered [17].

The SARS-CoV-2 Immunity and Reinfection Evaluation study in the United Kingdom, which researched COVID-19 infections, reinfections, and vaccine effectiveness, used a participant involvement panel to gain input on study design and strategies for engaging patients. Modifications to design, implementation, and study evaluation phases based on participant input resulted in a more than 15-fold increase in enrollment [18]. In neurology, a preference study of patients with Parkinson disease showed that pain levels, which were initially underemphasized as a clinical measurement, are a meaningful symptom for patients [19]. Subsequently, pain levels were used as a patient-relevant endpoint in a clinical trial with patients for whom motor symptom treatment alone did not meet patient needs [19,20]. In oncology, a patient input assessment tool developed during a phase I/II study on the myelofibrosis drug ruxolitinib was used to identify patient-relevant symptom reduction measurements in phase III studies, which ultimately played a role demonstrating efficacy for regulatory approval [21,22].

While tools for incorporating patient input in clinical trial development and continuous improvement already exist, there remains a lack of broad acceptance and use of these tools in practice [23]. Moreover, even when there is implementation of metrics to evaluate patient input in drug development, there is no one-size-fits-all approach across organizations [24]. To answer these challenges, collaborative participants considered the need for approaches described in the following section.

##### Validate Metrics and Establish a Framework for Defining Measures That Are Clinically Meaningful to Patients

Several methodologies, such as patient surveys, already exist for gathering patient insights to improve the design of clinical trials [24]. Patient surveys can be a relatively straightforward way of engaging patients while acquiring necessary and quantitative insight into their perspectives [25]. This feedback can be used to identify measurables that patients have flagged as clinically meaningful to them, which can then be more accurately incorporated into trial design. Proposed metrics have included advisory group support and involvement, patient retention, quality of evidence, and adherence rates. Patients should be engaged throughout the clinical trials process to ensure that accurate and ongoing feedback is received [16,26].

Pilot programs that build upon existing training opportunities would better equip researchers to use patient input methodologies, improving clinical trial design and enhancing patient inclusion. Establishing mentorship and other learning opportunities for researchers and clinicians to share approaches for using patient input and including patients as co-trainers and co-designers of pilot programs would further enable the integration of patient input throughout the therapeutic life cycle.

## Discussion

### Opportunities for Implementation

Despite evidence that incorporating patient input into R&D leads to higher-quality medicines that better align with patients’ needs and priorities and can enhance the efficiency and quality of clinical trials, scaling up the collection and use of patient input in decision-making remains a challenge. Deploying scientifically robust and evidence-based patient input methodologies—whether existing or novel—can help researchers and sponsors improve representation in clinical trials, ultimately improving patient outcomes while also meeting the needs of developers and regulators. However, even when a culture of “patients as partners” exists within an organization, action collaborative discussions emphasized that the science of patient input has not been consistently incorporated into study design, budgeting processes, or project timelines. Ultimately, patients should be viewed as equal and essential stakeholders in the clinical trials enterprise and should thus be considered in all steps of trial development.

FDA guidance for industry underscores the agency’s support for including patient experience data in clinical trials (Textbox 2) [27]. The 4 PFDD guidance frameworks have largely been well received by the field. For example, in response to *Patient-Focused Drug Development: Methods to Identify What Is Important to Patients* [28], the second guidance document, the Biotechnology Industry Organization wrote “BIO commends the FDA for working to ensure patient experiences are more systematically collected and used to inform the development and review of new therapies”[29]. Additionally, the National Organization for Rare Disorders said that “we appreciate that the emphasis of this draft guidance is on identifying what matters most to patients in terms of the disease and treatments” [30].

**Food and Drug Administration patient-focused drug development guidance series.**

Pursuant to the Food and Drug Administration Reauthorization Act of 2017 and the 21st Century Cures Act, the Food and Drug Administration is developing a series of four Patient-Focused Drug Development guidance documents to promote systematic approaches for collecting and using patient and caregiver input:

1. Patient-Focused Drug Development: Collecting Comprehensive and Representative Input (final guidance issued in June 2020) [8]. This guidance document illustrates methods for collecting patent data, with an emphasis on ensuring that the input collected is comprehensive and representative. A glossary of common terms used in Patient-Focused Drug Development was included as an appendix, which addresses concerns within the field about inconsistent terminology.
2. Patient-Focused Drug Development: Methods to Identify What Is Important to Patients (final guidance issued in February 2022) [28]. The second guidance document of the series provides insight on how to gather pertinent information from patients. The guidance helps direct stakeholders to gather information about symptoms, impacts of the disease, and other issues that are deemed important to patients. Collecting this information will help increase understanding of the disease and inform clinical trial design.
3. Patient-Focused Drug Development: Selecting, Developing, or Modifying Fit-for-Purpose Clinical Outcome Assessments (draft guidance issued in June 2022) [31]. The third guidance of the series aims to guide the methods for selecting, modifying, developing, and evaluating clinical outcome assessments (COAs). As listed within the guidance, sponsors can use high-quality measures of patients’ health for the following reasons: measuring what matters to patients; being clear about what was measured; appropriately evaluating the effectiveness, tolerability, and safety of treatments; and avoiding misleading claims.
4. Patient-Focused Drug Development: Incorporating Clinical Outcome Assessments Into Endpoints for Regulatory Decision-Making (draft guidance issued April 2023) [32]. The fourth guidance document of the series is focused on executing the use of COAs. Specifically, the guidance discusses methods to incorporate COAs into endpoints that can be used for regulatory decision-making. COAs typically do not provide direct insight on patient benefit, so this guidance aims to help ensure that COAs that provide indirect patient information correspond to relevant patient benefit.

### Building on Existing Frameworks

Available frameworks for the clinical research process offer practical approaches for engaging patients. For example, the PARADIGM consortium has developed a flexible monitoring and evaluation framework that incorporates tailored metrics to assess the influence of patient engagement on study relevance, efficiency, and outcomes [24]. This framework underscores the value of aligning metrics to ensure meaningful and context-sensitive evaluation of engagement practices. In another example, the Drug Information Association and the Tufts Center for the Study of Drug Development introduced tools to assess organizational preparedness for patient engagement [26,33]. This highlights the importance of structured strategies to translate goals into actionable practices. Similarly, the Clinical Trials Transformation Initiative’s (CTTI’s) Patient Engagement Collaborative demonstrates how multistakeholder partnerships can foster dialogue to improve engagement throughout the medical product life cycle. CTTI has advanced efforts to integrate patient input into decision-making processes and regulatory practices by providing a platform for patients, regulators, and other stakeholders to share perspectives and resources [34]. Additionally, companies have codeveloped frameworks with patient advisors on how to capture and assess patient input throughout the medical product life cycle [35]. More is needed to build upon, scale, and incentivize the implementation of such frameworks for patient engagement and advance the field toward systemwide application of the science of patient input.

### Demonstrating Return on Investment

The impact of patient input on clinical research is expected to continue to unfold over time, necessitating ongoing evaluation and refinement of methodologies. Short-term effects might be observable in improved trial approval rates, retention, and accrual, while the long-term benefits could manifest in more meaningful trial endpoints and the development of valuable medications. However, the absence of control arms specifically designed to evaluate patient input outcomes presents a challenge in directly attributing improvements to patient input. Nonetheless, early benefits in trial conduct suggest that patient input can enhance the research process.

The available literature regarding return on engagement of gathering patient input and how patient input impacts the ultimate cost of therapeutic development is limited. Filling those gaps is an important step toward demonstrating the value of patient input to drug developers. In one example, a team of sponsors, patient groups, investigators, and health economists developed an economic model for return on investment from patient input in phase II and III trials. The model suggested that using patient input can lead to financial benefits associated with avoiding protocol amendments and improving patients’ study experience [36]. In addition, health technology assessment agencies are increasingly interested in using patient experience data for decision-making, including information on medical interventions, quality-of-life measures, and value assessments that inform reimbursement decisions [37]. While it stands to reason that patient-centric trials would be more successful in recruitment and retention, thus supporting more robust outcomes at a faster rate, consistent practice and rigorous data collection will be needed to provide better quantification of the economic impact of patient input.

### Integrating Patient Input Into “Business as Usual”

Integrating patient input into clinical trial standard operating procedures requires not only a modest investment of capital but, more importantly, a significant commitment to change management. Evidence-based patient insights help sponsors identify and address pain points in protocol design, recruitment, and retention, making trials more efficient and representative. To fully integrate patient input throughout an organization—from the R&D teams to clinical trial sites—requires leadership as well as systematic updates to workflows and training, allocation of resources, and shared accountability.

To achieve this, sponsor companies must empower key decision makers to champion a cultural shift that cascades throughout clinical operations. This shift should ensure that patient input is systematically collected, analyzed, and made available to teams as a standard resource—either as a curated library of insights or proactively gathered ahead of need—so that it informs decisions at critical points in the R&D cycle. Crucially, these insights must be generated during the process, not retrofitted after decisions are made, to maximize their impact and enable true cocreation of R&D programs with embedded, inherent patient input.

Practical next steps for industry may include (1) collaborating with leading patient advocacy organizations to create centralized, therapeutic-area–specific patient councils that generate reusable patient preference data—reducing the need for each sponsor to start from scratch; (2) partnering with patient engagement experts to establish a core, evidence-based set of principles to guide the use of patient input for improving trial design and outcomes; (3) establishing a cross-industry working group to collate, refine, and regularly update best practices for patient engagement, particularly those that are therapeutic-area agnostic; (4) integrating existing publicly available tools—such as those produced by TransCelerate, CTTI, Patient-Centered Outcomes Research Institute, and others—directly into trial design workflows and training programs; and (5) establishing common metrics and key performance indicators to assess improvements in recruitment speed, participant representation, and retention as patient engagement practices mature.

Patients and advocacy groups must likewise invest time and resources to strengthen their partnerships with industry. This may include cultivating informed patient representatives who can clearly articulate the lived experience, treatment journey, and burden of disease, and serve as a reliable resource for sponsors to engage. As these relationships mature, and as patients and advocacy groups demonstrate their value alongside sites, Contract Research Organizations, and other service providers, sponsor organizations will benefit by accelerating protocol optimization, improving trial feasibility assessments, and institutionalizing the science of patient input as a core component of clinical development.

### Engaging Patients as Partners

Enhancing the capacity to support patient advocates is a crucial area for development as well. Each of the approaches outlined in this paper requires the engagement of stakeholders across the entirety of the patient journey, including patients, families, caregivers, clinical teams, trialists, sponsors, digital health companies, EHR vendors, regulators, and data scientists. Addressing barriers that impede patient involvement in advocacy and research, including economic barriers and lack of accessible health care, will take concerted efforts. Such an approach is essential for harnessing the full potential of patient input in shaping the future of clinical trials and health care innovation.

Successful patient partnerships should include representation of the populations affected by the disease. People who are underrepresented in clinical research—including people with comorbidities and those who are not well represented in patient organizations—can be reached through more inclusive measures and methodologies that integrate patient-centered insights on disease burden. This inclusivity would help ensure that the outcomes and recommendations derived from patient input reflect the varied experiences and needs of people from across different communities. Moreover, embracing inclusive patient partnerships fosters a more holistic understanding of the disease landscape. While challenges remain, collaborative cross-sector efforts around transparency in decision-making and the application of insights gained from patient input offer an opportunity to improve health care in a way that is meaningful to the most important stakeholders of all—the patients.

## Appendix

Multimedia Appendix 1 Research priorities and opportunities.

Multimedia Appendix 2 Understanding the patient experience over the course of a disease or condition.

Multimedia Appendix 3 Barriers and potential actions for understanding the patient experience over the course of a disease or condition.

Multimedia Appendix 4 Capturing patient perspectives and priorities on benefits and risks of medical product use.

Multimedia Appendix 5 Incorporating patient input into continuous improvement of clinical trial design and development.

## Abbreviations

CTTI: Clinical Trials Transformation Initiative
EHR: electronic health record
FDA: US Food and Drug Administration
PFDD: Patient-Focused Drug Development
R&D: research and development
