# Supplementary material for: Advancing the Science of Patient Input in Drug Research and Development
Source: J Particip Med. 2026 May 1;18:e74436. doi: 10.2196/74436 (PMC13179488; doi:10.2196/74436)
Supplement: Multimedia Appendix 1 [file jopm_v18i1e74436_app1.docx]

# Supplemental Methods: Information-Gathering

Following the 2018 National Academies public discussion-based workshop on *Advancing the Science of Patient Input in Medical Product R&D: Towards a Research Agenda*, collaborative participants designed and administered a set of questions via email in January 2019 to gather information on high priority areas which, if effectively addressed through targeted research and/or funding, would significantly advance the science of patient input. Collaborative participants circulated questions to 8 individuals (3 respondents) and 17 organizations (5 respondents); respondents were offered the option of responding in writing or through informal phone interviews. In addition, the Professional Society for Health Economics and Outcomes Research (ISPOR) circulated the series of questions to 6 relevant member special interest groups (30 respondents).

Responses were collected and organized based on the following cutoffs: 1) Gap/barriers selected as high priority by more than half of respondents; and 2) Gap/barriers selected as high priority by more than half of ISPOR or email respondents (see Box 2).

The collaborative then held a meeting on July 30, 2019, to discuss research gaps that should be filled and barriers that should be overcome to advance the science of patient input (see Supplemental Table 1 for list of meeting participants). During the meeting, participants used dot voting to indicate which gap/barriers they considered to be high priority areas for future research. Participants discussed gaps/barriers they considered to be high priority in small working groups. The working groups shared a summary of their discussions in a plenary session and through written responses.

**Recipient Organizations**

American Diabetes Organization; American Heart Association; American Society of Clinical Oncology; Biotechnology Innovation Organization (BIO); Clinical Trials Transformation Initiative (CTTI); Diatribe Foundation; Drug Information Association (DIA); Epilepsy Foundation; Every Life Foundation; International Foundation for Autoimmune & Autoinflammatory Arthritis; International Society for Quality of Life Research; Michael J. Fox Foundation; Milken Institute FasterCures; National Multiple Sclerosis Society; National Organization for Rare Disorders’ Pharmaceutical Research and Manufacturers of America (PhRMA); Transcelerate.

ISPOR Respondent Member Group Affiliations

Respondents may belong to more than one member group, 30 total responses received

*Member Group*: Patient Centered Special Interest Group, 11 respondents

*Member Group*: Patient Representatives Roundtable , 9 respondents

*Member Group*: Clinical Outcomes Assessment Special Interest Group, 6 respondents

*Member Group*: Health Preference Special Interest Group, 6 respondents

*Member Group:* Real World Evidence Special Interest Group, 4 respondents

*Member Group*: Statistical Methods in HEOR Special Interest Group, 1 respondent

## **Materials and Questions Shared with Recipients in 2019**

**Gaps and Barriers to Advancing the Science of Patient Input**

On May 9, 2018, the National Academies Forum on Drug Discovery, Development, and Translation (the Forum) hosted a workshop to examine gaps in knowledge and other barriers that hinder the advancement of a science of patient input in medical product research and development. For more information on the workshop, please find a proceedings-in-brief here:

<http://nationalacademies.org/hmd/Reports/2018/advancing-the-science-of-patient-input-in-medical-product-r-and-d-proceedings-in-brief.aspx>

The following tables provide a list of gaps/barriers organized by topic that were raised by workshop participants. Some gaps/barriers may be addressed by current initiatives, while others may not be studied or researched at all. Under the aegis of the Advancing the Science of Patient Input Action Collaborative^^[[1]](#footnote-1)^^ (the collaborative), an ad hoc activity associated with the Forum, collaborative participants have set out to identify and prioritize critical gaps in the knowledge base and other barriers that impede progress for the field.

Your input and perspective will help inform the thinking of the collaborative participants as they work to develop a research agenda for addressing key gaps and barriers, which could help better align medical product discovery, development, and regulation with patient preferences for disease management and treatment.

**Request:**

Please review each topic and select the top 3-5 gaps/barriers (within each topic) that you feel are most critical to prioritize (e.g. gaps/barriers that should be tackled first and/or would have the most impact if effectively addressed).

Feedback on the gaps/barriers (is there anything we missed?), reasons why you selected particular gaps/barriers, connections observed across topics, and/or possible approaches/solutions are welcome in the comments section.

Your name:

Organization:

| **Topic 1: Understanding the Patient Experience with a Disease or a Medical Condition**  ***Please select the top 3***$\boldsymbol{-}$***5 gaps/barrierswithin Topic 1 that you feel are most critical to prioritize*** |
| --- |
| Methods  ☐ Clinical data collection does not generate information that accurately describes the patient holistically  ☐ Challenge to define the right questions to support meaningful study re: patient input  ☐ Lengthy process of establishing natural history and managing patient expectations at early stages of research  ☐ Lack of data that reflect in-depth capture of patient “experience,” which includes personal and clinical experience  ☐ Lack of defining quality standards and rigor for quantitative and qualitative patient input data  ☐ Approaches/methods/metrics needed to quantify and measure psychological burden on patients  Data  ☐ Databases not built for patient input purposes  ☐ Patient perspective and/or patient input information is not incorporated/integrated into electronic health records (EHRs)  ☐ Electronic medical records (EMRs) are not integrated and/or aggregated with claims data  ☐ Challenge to incorporate learnings from patient input in care/research system that may inform development of new treatments (i.e. Implementation of a Learning Health System and Care-based Research)  ☐ Lack of data on use of medicines/available treatments in the real world  Patient Populations  ☐ Lack of patient input regarding healthcare disparities  ☐ Variable understanding of unmet medical need (by all stakeholders)  ☐ Challenge to reach/engage some patient populations with specific/rare diseases  ☐ A national registry does not exist for every disease for which there is an unmet need  PROs/PCOs  ☐ Existing patient reported outcomes (PROs) data sets may not capture baseline/meaningful difference for a given patient  ☐ Symptom burden from the perspective of the patient that maps to disease trajectory is not captured in a standardized way  ☐ Challenge to maintain patient privacy and decrease the feeling of invasion (for the patient) when using data collection devices in the patient’s home |

| **Topic 2: Capturing Patient Perspectives and Preferences on Benefit–Risk**  ***Please select the top 3***$\boldsymbol{-}$***5 gaps/barrierswithin Topic 2 that you feel are most critical to prioritize*** |
| --- |
| Methods  ☐ Lack of understanding/methodologies for differentiating between measurement error vs heterogeneity of data due to heterogeneity of patients  ☐ Lack of understanding/methodologies in quantifying change in benefit/risk tolerance and/or uncertainty tolerance over time of age/disease progression  ☐ Lack of understanding/approaches/methods for Institutional Review Boards (IRB) to integrate patient perspective  ☐ Lack of knowledge regarding the ability of patients to predict future benefit-risks  ☐ Challenge of identifying the right point to have a conversation with the patient on benefit/risk preferences in patient treatment pathway  Data  ☐ Underuse of existing large data sets  Patient Populations  ☐ Lack of understanding/methodologies for obtaining input from patients who cannot directly express their preferences (e.g. pediatric patients, cognitively impaired patients, such as patient with dementia or Alzheimer’s)  ☐ Challenge in identifying patient subgroups willing to accept higher risk  ☐ Lack of perspectives from patients with multiple comorbidities  ☐ Lack of knowledge regarding how patients with different diseases/conditions deal with uncertainty  ☐ Lack of knowledge/methods for understanding how certain patient populations perceive and understand information differently due to cultural or social differences  ☐ Better understanding needed when it comes to risk perception on the part of patients versus other stakeholders (e.g. pharma, clinicians, regulators); inadequate consideration of patient perceptions as compared to other stakeholders  PROs/PCOs  ☐ Lack of information on the current state of funding for the science of patient input/preferences |

| **Topic 3: Incorporating Patient Input in Clinical Trial Development and Continuous Improvement**  ***Please select the top 3***$\boldsymbol{-}$***5 gaps/barrierswithin Topic 3 that you feel are most critical to prioritize*** |
| --- |
| Methods  ☐ Lack of a standard framework and metrics for measuring trial participant experience  ☐ Challenges with selection bias, survey fatigue, loss to follow up, and survivor bias  ☐ Challenge of defining and validating meaningful measures  ☐ Lack of metrics to determine value of patient input in protocol development  ☐ Lack of understanding/methods regarding how patient input impacts enrollment and retention  ☐ Need for methodologies to carry out necessary statistical modeling for incorporating patient input  ☐ Challenge when it comes to repeated measures and sampling issues  Data  ☐ Challenge of documenting patient input in claims data  Patient Populations  ☐ Challenge in acquiring information from patients if the patients are cognitively impacted by their disorder and/or patient populations that cannot directly express their preferences  ☐ Lack of knowledge/methods for soliciting caregiver and/or family experiences during the trial process, as well as the impact of trial participation on these stakeholders  ☐ Lack of methods/approaches for better representing the broader patient community  ☐ Lack of statistical models for data segmentation when eligibility is intentionally broadened  ☐ A national registry does not exist for every disease for which there is an unmet need  ☐ Lack of understanding/methodologies for obtaining appropriate consent from patient populations that cannot directly express their preferences (e.g. pediatric patients, cognitively impaired patients, such as patient with dementia or Alzheimer’s)  ☐ Lack of knowledge/methods for understanding how certain patient populations perceive and understand information differently due to cultural or social differences  Change Management  ☐ Need approaches to encourage a culture of incorporating patient input early on in trial design  ☐ Lack of training for scientists to understand the value proposition for gathering and using patient input  ☐ Lack of training/preparedness on the part of patients/patient groups to provide expert input  ☐ Need for FDA to take the lead in determining standards of patient input in trials  ☐ Reputation may be a barrier for engaging patients at new clinical sites or sites with poor performance recruiting |

**Comments:**

- Is there anything we missed?
- Why did you select particular gaps/barriers?
- Any connections observed across topics?
- Any proposed approaches/solutions?

1. The collaborative is an ad hoc activity associated with the Forum on Drug Discovery, Development, and Translation (the Forum) at the National Academies of Sciences, Engineering, and Medicine (the National Academies). The work of the collaborative does not necessarily represent the views of any one organization, the Forum, or the National Academies and is not subjected to the review procedures of, nor is it a report or product of, the National Academies. [↑](#footnote-ref-1)
