# Supplementary material for: Advancing the Science of Patient Input in Drug Research and Development
Source: J Particip Med. 2026 May 1;18:e74436. doi: 10.2196/74436 (PMC13179488; doi:10.2196/74436)
Supplement: Multimedia Appendix 2 [file jopm_v18i1e74436_app2.docx]

Multimedia Appendix 2

Table S1. Understanding the patient experience over the course of a disease or condition.

To examine some of the different types of patient input that can be collected and used to better understand the patient experience over the course of a disease or condition, collaborative participants assembled information from 1) a public National Academies workshop on *Advancing the Science of Patient Input in Medical Product R&D: Towards a Research Agenda*, 2) responses to a set of questions sent to representatives from non-profit associations, biopharmaceutical trade organizations, patient groups, and relevant member special interest groups for the Professional Society for Health Economics and Outcomes Research (ISPOR), and 3) an in-person cross-sector meeting of action collaborative participants to discuss research priorities and opportunities to advance the science of patient input. The following table summarize some of the potential applications, methods, and data sources for different types of patient input that were discussed at the meeting.

Data sources may include but are not limited to:

- Patient preference studies
- Postmarket studies (e.g., patient outcome/patient satisfaction studies)
- Epidemiology study data
- Clinical effectiveness research
- Behavioral data
- Patient registries
- Biobanks
- Genomic data
- Electronic Health Records (EHRs)
- Claims data
- Medical reports
- Patient reported outcomes (PROs); PROs linked to claims
- Patient surveys and questionnaires
- Patient interviews
- Patient and caregiver listening sessions/focus groups
- Case studies
- Clinician-reported data
- Passive technology data capture (e.g., in-home devices, wearables, and other digital health technologies)
- Social media

| Patient Input on the Natural History of a Disease or Condition | |
| --- | --- |
| Applications | **Methods** |
| Tool creation   - Inform biomarker development - Support the establishment and modification of endpoints that are meaningful for patients throughout the course of a disease or condition   Healthcare delivery decision-making   - Inform treatment strategies (e.g., timing of treatment) - Contribute to value measures for medical interventions - Help link patient phenotype and genotype - Support the tracking of disease development over time - Helps clinicians identify pre-diagnosis symptoms/signs of disease   Drug R&D decision-making   - Inform all stages of drug development, from pre-discovery through post-market surveillance - Support the business case for drug development - Inform timeframes for conducting a study - Support selection of research priorities and therapeutic approaches - Inform inclusion/exclusion criteria for trials conducted at different stages of disease - Inform selection of patient populations for clinical trial enrollment - Inform pre-discovery insights through post-market surveillance - Inform comparing treatment efficacy; potential control arm for small population studies   Other   - Provide researchers, sponsors, and regulators with a better understanding of the patient journey over time | - Social listening/social interviews - Patient surveys - Collection of quantitative and qualitative data quality of life (QOL) measures - Collection of data from exam based natural history studies - Collection of data from new technology tools (e.g. wearables and carriables) - Avatar-assisted journaling |

| Available Treatment Options and Unmet Medical Need | |
| --- | --- |
| Applications | **Methods** |
| Tool creation   - Guide modifications of endpoints   Inform healthcare delivery decision-making   - Allow for better measurements of treatment combinations to include complementary and alternative therapies - Determine a credible, cost effective and patient-friendly treatment protocol for patients with co-morbidities, using both conventional and unconventional approaches   Address Unmet Medical Need   - Avoid the development of drugs that do not address patient’s needs and clinician’s desires - Direct research priorities   Other   - Guide formulary committee decisions - Inform investment and discussions about value of medicines/treatments | - Co-created surveys with patients; interviews with patients and physicians - Development of guidelines on how to curate available data options - Employment of approaches that refine the research question to include when, how, for whom, and by whom data are used - Quantitative and qualitative methods |

| Burden of Living with a Disease or Condition | |
| --- | --- |
| Applications | **Methods** |
| Inform drug R&D decision-making   - Understand the burden of symptoms; the importance to the patient may change over time - Inform preclincial and clinical research questions - Inform endpoint development and selection - Inform the design of a delivery method - Inform product design, study design, preclinical research - Inform delivery methods/formulation   Address Unmet Medical Need   - Move from assumptive burden to actual burden and identify unmet need (aspect of health) that needs to be addressed by the medical product   Other   - Decrease the burden of the patient’s disease - Improve understanding of patient disease burden on the part of researchers | - Patient surveys - Longitudinal observational studies - Use of tools to capture “this is what is happening to me; here is how I am managing it”; non-financial/opportunity costs - Development of a web source that constantly updates to stay current - Tapping into existing communities in healthcare settings - Social media mining - Patient journey mapping- from multiple stakeholders (caregiver, patient, clinician) - Collection of qualitative information from patient and/or caregiver focus and working groups - Landscape review on unmet needs and develop methods with those needs in mind - Qualitative social networking |
