# Supplementary material for: Advancing the Science of Patient Input in Drug Research and Development
Source: J Particip Med. 2026 May 1;18:e74436. doi: 10.2196/74436 (PMC13179488; doi:10.2196/74436)
Supplement: Multimedia Appendix 3 [file jopm_v18i1e74436_app3.docx]

Multimedia Appendix 3

**Table S1.** Barriers and potential actions for understanding the patient experience over the course of a disease or condition.

| Stakeholder | | Barriers | Potential actions | Benefits of taking action |
| --- | --- | --- | --- | --- |
| **Understanding the patient experience over the course of a given disease or medical condition** | | | | |
|  | Sponsors (private, federal, and nonprofit funders of trials) | - Real and perceived barriers to engaging patients within a regulated environment - Lack of organizational and management change | - Leadership prioritize patient engagement - Incorporate patient input metrics - Develop and implement patient engagement strategies and training programs throughout the drug R&D^a^ life cycle. | - Continuing evolution of patient-sponsor partnership - Increased likelihood of successful development outcomes - Development of more meaningful endpoints for patients |
|  | Study staff (including principal investigator, health care professionals, and providers) | - Difficulty capturing heterogenous patient experiences - Inability to effectively incorporate patient input in a way that informs clinical practice | - Customize approaches for engaging disease-specific or sociocultural communities - Define quality standards and rigor for quantitative and qualitative data - Use tools for real-time data capture over the course of the patient journey | - Informing clinical trial development, product design, trial design, and clinical research questions - Insights on health care disparities for specific patient populations or communities - Development of more meaningful endpoints for patients |
|  | Health care facilities and sites | - Limited access to patient history across sites | - Integrate electronic health records with patient-reported outcomes - Build partnerships to share data across facilities - Implement a learning health system and care-based research | - More comprehensive understanding of patient journey across multiple sites |
|  | Patient advocacy groups | - Cultural, social, and economic differences across patient populations and communities | - Tailor outreach strategies and educational programs that meet the specific needs and preferences of particular patient populations | - Provide a better understanding of the full patient journey - Have more meaningful input into sponsor and site engagements |
|  | Regulators | - Difficulty incorporating heterogeneous patient feedback in decision-making | - Generate guidances and use cases to illustrate how patient-centric goals can be incorporated into regulatory review - Continue development of guidelines for patient engagement - Facilitate direct engagement with patients and caregivers | - Increased trust in the regulatory process - Provide clearer and more targeted guidance for sponsors and sites on how to compliantly interact with patients and PAGs^b^ |
|  | Digital health technology companies | - Variation in patient internet access and technology literacy - Challenges working across multiple sources of data | - Increase patient input and engagement throughout technology development, validation, and implementation - Develop patient and caregiver tool kits for providing input that meets medical needs, reduces burden, and positively impacts the health care experience | - Generation of information that more accurately describes the patient experience - Enhanced trust in technology-driven health solutions - Better fit-for-purpose tools that reduce burden on sites and patients |
|  | Electronic health record vendors | - Lack of clarity on the types of patient input data that are relevant or needed - Lack of a comprehensive view of databases | - Create interoperable and patient-centered industry standards for core technologies | - Patient input data become more interchangeable among systems and interfaces, enabling a comprehensive and correct view of medical history |
|  | Data scientists | - Limited access to patient input data - Lack of standardized methods for quantifying and incorporating patient input data in analyses - Difficulty in aggregating data from heterogeneous sources | - Expand data-sharing practices using methods that maintain patient data security - Publicly discuss methods or metrics to identify gaps and agree on priorities for incorporating these data going forward - Use advanced analytics (AI^c^ and machine learning) to integrate diverse datasets | - Improved patient input metrics and insights - Opportunity to collect and analyze patient input longitudinally - Could lead to new research and publications that promote and justify the investment in patient input |
|  | Journal editors and reviewers | - Limited awareness or understanding on the importance and methods of collecting and incorporating patient input data - Difficulty identifying reviewers with expertise in the science of patient input - “Traditional” criteria for scientific review may not capture emerging approaches for collecting and using patient input in drug R&D | - Disseminating information to journals, including through trainings and convenings, that communicates the importance of patient input in medical product development - Identify and train reviewers with expertise in the science of patient input, including more patients and patient advocate reviewers - Define expectations for submitted manuscripts that incorporate patient input | - Increased emphasis on the importance of patient input research and encouraging the inclusion of patient perspectives - Helps ensure that research findings are communicated in a patient-centered manner, promoting wider dissemination and understanding of the research |
| **Capturing the patient perspectives and priorities on benefit-risk** | | | | |
|  | Sponsors (private, federal, and nonprofit funders of trials) | - Lack of patient-centered endpoints | - Partner with patient advocacy groups to prioritize patient-centered endpoints and endpoints that align with patient values and health outcomes that matter most to them | - Clinical research data retain existing rigor for regulatory approval but better align with patient community needs |
|  | Study staff (including principal investigator, health care professionals, and providers) | - Lack of patient-centered endpoints | - Collect burden and quality-of-life measures that illuminate the patient experience | - Improved *accrual and* retention of *future* trial participants |
|  | Health care facilities and sites | - Mistrust and distrust from specific patient communities | - Tailor outreach to patient communities to better understand patient concerns and build rapport over time | - Improved recruitment, retention, and participant experience |
|  | Patient advocacy groups | - Challenge to reach or engage certain patient populations (eg, patients living with rare diseases or conditions) | - Develop outreach campaigns that are designed to reach populations affected by diseases or conditions, using platforms and channels that are most effective for engaging those communities | - Inform critical intervention points and treatments relevant to patients - Broaden health care options and access for constituent populations |
|  | Regulators | - Difficulty in balancing regulatory requirements and patient-centric goals | - Disseminate information about emerging methods and guidelines for gathering patient input to patient groups and researchers | - More transparency on how patient input informs medical product decision-making |
|  | Electronic health record vendors | - Inconsistently displayed patient input data in EHRs^d^ - Difficulty in capture of patient-reported outcomes in EHRs - Lack of incentives to update data and information | - Offer patient-friendly, consistent data fields - Build tools to transform data into information that are relevant for patients, study sites, clinicians, sponsors, and regulators - Provide resources and guidances to promote continuing data collection, storage, and maintenance | - Data presented in formats that allow patients and health care professionals to understand what information means in the context of diagnosis and treatment |
|  | Journal editors and reviewers | - Journal papers on patient input and medical product development often not accessible for patients and caregivers | - Generate plain language summaries alongside primary research publications | - Evidence-based materials that can be understood and useful to a broader audience, including patients and caregivers |
| **Incorporating patient input into clinical trial design and continuous improvement** | | | | |
|  | Sponsors (private, federal, and nonprofit funders of trials) | - Difficulty in meeting patient expectations (eg, patient input may not benefit an individual directly) - Long trial durations | - Clearly communicate the potential benefits of patient input, both for individual patients and the broader community - Simplify and streamline clinical trial protocols to reduce trial duration and patient burden while maintaining scientific rigor | - More informed investment and discussion on the value of treatments - Faster, better, and more frequent feedback from patients that can help industry improve the patient medical experience |
|  | Study staff (including principal investigator, health care professionals, and providers) | - “Traditional” mindsets that do not involve study cocreation or collaboration with patients | - Facilitate conversations between study staff and patient groups to establish shared baselines, metrics, and study design | - Reduced tension and friction in provider-patient relationships and more collaborative health care relationships |
|  | Health care facilities and sites | - Lack of trust or established relationships between a study team and potential trial participants | - Integrate principal investigators and site teams that self-identify with local communities | - Enhanced representation of patient voices throughout clinical trial design and execution - Higher comfort level and trust with HCPs^e^ |
|  | Patient advocacy groups | - Limited resources for patient input | - Provide funding and support for training on fundraising strategies to help secure resources for patient-centered research | - Support credible, cost effective, and patient-friendly treatment protocols, particularly for complex patient populations with comorbidities |
|  | Regulators | - Lack of insight on the variety of methods that sponsors use to collect patient input throughout the drug R&D process | - Enhance communication between regulators and sponsors on emerging methods for collecting patient input | - Generate more consistent patient-centered evidence to inform regulatory decision-making in drug development and postapproval programs |
|  | Digital health technology companies | - Complexity and nuances associated with enabling technologies that are fit for purpose at the trial, site, or patient population level - Rapid changes in technology outpacing validation processes | - Incorporate patient input into clinical data collection, including environmental health and safety data - Establish common data governance frameworks - Convene stakeholders to share lessons learned on technology usage and discuss emerging validation strategies | - New models for in-home data collection - Developing sound practices and standards for patient interactions with digital health tech in a rapidly evolving technical climate |

^a^R&D: research and development.

^b^PAGs: patient advocacy groups.

^c^AI: artificial intelligence.

^d^EHRs: electronic health records.

^e^HCPs: health care providers.
