# Supplementary material for: Advancing the Science of Patient Input in Drug Research and Development
Source: J Particip Med. 2026 May 1;18:e74436. doi: 10.2196/74436 (PMC13179488; doi:10.2196/74436)
Supplement: Multimedia Appendix 4 [file jopm_v18i1e74436_app4.docx]

Multimedia Appendix 4

Table S1. Capturing patient perspectives and priorities on benefits and risks of medical product use.

To examine some of the different types of patient input that can be collected and used to better capture patient perspectives and priorities on benefits and risks of medical product use, collaborative participants assembled information from 1) a public National Academies workshop on *Advancing the Science of Patient Input in Medical Product R&D: Towards a Research Agenda*, 2) responses to a set of questions sent to representatives from non-profit associations, biopharmaceutical trade organizations, patient groups, and relevant member special interest groups for the Professional Society for Health Economics and Outcomes Research (ISPOR), and 3) an in-person cross-sector meeting of action collaborative participants to discuss research priorities and opportunities to advance the science of patient input. The following table summarize some of the potential applications, methods, and data sources for different types of patient input that were discussed at the meeting.

Data sources may include but are not limited to:

- Patient preference studies
- Longitudinal studies to measure tolerability
- Clinical trials that include the use of tools for using the Patient-Reported Outcomes (PROs) version of the Common Terminology Criteria for Adverse Events and other PRO measures
- Claims data
- Medical reports
- Patient interviews
- Patient and caregiver listening sessions/focus groups
- Behavioral data
- Passive technology data capture (e.g., in-home devices, wearables, and other digital health technologies)
- Social media

| Benefit and Risk Tradeoffs | |
| --- | --- |
| Applications | **Methods** |
| Inform drug R&D decision-making   - Use towards development planning (e.g., target selection, endpoint definition) - Inform product level decisions during development (go/no-go at various phases) - Product level decisions (e.g. formulation, dosing, submission) - Help define research questions - Inform study design, including subpopulations to include - Inform the need and design for post-market studies   Other   - Inform regulatory decision-making - Predict investment - Identify subgroups of patients willing to take on higher risk - Ensure understanding of patient viewpoint on benefits and risks - Inform regulatory decision making | - Benefit/risk analysis - FDA patient focused drug discovery (PFDD) meetings and facilitated regulatory conversations - Use of patient preference tools - Patient surveys - Patient testimonials - Collection of caregiver input as a proxy for patient input - Inclusion of patient perspective/preference in a benefit/risk framework - Use of shared decision-making tools |

| Risk Tolerance | |
| --- | --- |
| Applications | **Methods** |
| Tool creation   - Inform tool development for how patients and caregivers evaluate risk   Inform healthcare delivery decision-making   - Better match treatments for patients based on a person’s risk profile   Inform drug R&D decision-making   - Link primary and secondary endpoints to values of patients   Other   - Determine real life impact- social determinants (priorities taking other life factors into account) | - Patient surveys - Patient testimonials - Adoption of methods used to assess financial risk |

| Expectation of Benefit | |
| --- | --- |
| Applications | **Methods** |
| Tool creation   - Develop surrogate endpoints - Differentiate between function versus improvement of symptom or biomarker - Appropriate endpoint(s) selection   Inform drug R&D decision-making   - Help define what patients consider an unmet medical need - Inform on research priorities and questions - Inform decisions treatment options to pursue - Inform the selection of outcomes/study endpoints that are meaningful for patients (e.g., survival, extension of life, quality of life) - Inform design of the study - Inform on interpretation of study results and what would be considered meaningful benefit for patients - Help determine where resources should be spent throughout the R&D process | - Qualitative methods, potentially based on quantitative methods (i.e. sentiment analysis) - Take generalized concept and operationalize specifically to disease/population; - Qualitative interviews, focus groups - Patient surveys - Embed questions about minimum expectations of events into larger data gathering efforts |
