# Supplementary material for: Advancing the Science of Patient Input in Drug Research and Development
Source: J Particip Med. 2026 May 1;18:e74436. doi: 10.2196/74436 (PMC13179488; doi:10.2196/74436)
Supplement: Multimedia Appendix 5 [file jopm_v18i1e74436_app5.docx]

**Multimedia Appendix 5**

**Incorporating patient input into continuous improvement of clinical trial design and development.**

To examine some of the different types of patient input that can be collected and used to incorporate patient input into continuous improvement of clinical trial design and development, collaborative participants assembled relevant information from 1) a public National Academies workshop on *Advancing the Science of Patient Input in Medical Product R&D: Towards a Research Agenda*, 2) responses to a set of questions sent to representatives from non-profit associations, biopharmaceutical trade organizations, patient groups, and relevant member special interest groups for the Professional Society for Health Economics and Outcomes Research (ISPOR), and 3) an in-person cross-sector meeting of action collaborative participants to discuss research priorities and opportunities to advance the science of patient input. The following table summarize some of the potential applications, methods, and data sources for different types of patient input that were discussed at the meeting.

Data sources may include but are not limited to:

- Patient preference studies
- Postmarket studies (e.g., patient outcome/patient satisfaction studies)
- Behavioral data
- Patient registries
- Electronic Health Records (EHRs)
- Claims data
- Medical reports
- Patient surveys and questionnaires
- Patient interviews
- Patient and caregiver listening sessions/focus groups

| Trial Participant Experience | |
| --- | --- |
| Applications | **Methods** |
| Inform healthcare delivery decision-making   - Understanding how trial design affects recruitment, enrollment, retention, time, and cost - Continuous evaluation, learning, improvements that inform downstream activities - Patient experience in non-measurable areas (i.e. blood draw) - Inform risk-based monitoring decisions   Inform drug R&D decision-making   - Inform potential protocol amendments - Inform future study design and processes - Faster enrollment of patients into trials - Increased patient retention - Reduced costs - Increased researcher understanding and appreciation of realities of human experience - Patient engagement impact on trial itself; design vs. execution comparison | - Use of product lifecycle management software tools - Engagement with patient advisory boards - Feedback/exit interviews from trial participants (patient retention and adherence rates) - Use of adaptive trial design - Employment of continuous trial checkpoints/check-ins (patient retention and adherence rates) |

| Protocol Development | |
| --- | --- |
| Applications | **Methods** |
| Tool creation   - Endpoints that are sensitive to capture incremental improvements, capturing endpoints that reflect symptoms that matter to patients and also have biological impact - Mechanism for measuring the cost savings that can result when patients are involved in the protocol development process   Inform drug R&D decision-making   - Establish inclusion/exclusion criteria - Primary and secondary endpoints - Critical to inform protocol design; earlier is better at point of hypothesis - Inform trial procedures and feasibility, what is tolerable to patients, impact on life - Measure the patient experience in a clinical trial. - Increase the speed of protocol development and recruitment - Return on Engagement (ROE) - Stated preference versus experiential preference - Identify appropriate comparators for other users and label expansion   Other   - Inform company policies that involve patient input as the expectation (if not a requirement) | - Protocol co-design sessions with patient representatives (advisory group support and involvement) - Day-long key opinion leader (KOL) and patient opinion leader (POL) meetings for protocol co-design (advisory group support and involvement) - Seeking feedback from patients, patient group leadership, trained research advocates, and patient advisory boards - Initial qualitative/quantitative methods, use surveys to get to a representative group, online and social media; think through methods specific to disease areas - Mixed methods to potentially identify off label use through electronic health records and validate by interviewing/surveying patients |

| Recruitment, Enrollment, and Retention | |
| --- | --- |
| Applications | **Methods** |
| Inform healthcare delivery decision-making   - Inform doctors of the need to learn more about clinical trials so as to better inform their patients   Inform drug R&D decision-making   - Minimize failures in screening - Better trial design, recruitment, enrollment, and retention processes - Allow for faster and more efficient recruiting; minimize screening failure and dropouts - Identify patients for trials - Inform study design/protocol development - Inform potential protocol amendments   Other   - Improved data collection (efficiency), lower costs | - Using patient registries and clinical registries to help select eligible trial participants; proactively reaching out to potential participants - Extension studies - Analysis of quantitative data and mixed data |
